# Routine clinical cardiovascular magnetic resonance in paediatric and adult congenital heart disease: patients, protocols, questions asked and contributions made

**DOI:** 10.1186/1532-429X-10-46

**Published:** 2008-10-17

**Authors:** Sohrab Fratz, John Hess, Annika Schuhbaeck, Christine Buchner, Eva Hendrich, Stefan Martinoff, Heiko Stern

**Affiliations:** 1 Department of Paediatric Cardiology and Congenital Heart Disease, Deutsches Herzzentrum München an der Technischen Universität München, Munich, Germany; 2 Department of Radiology, Deutsches Herzzentrum München an der Technischen Universität München, Munich, Germany

## Abstract

**Background:**

Cardiovascular Magnetic Resonance (CMR) of patients with congenital heart disease (CHD) has become routine clinical practice. However, existing CMR protocols focus predominantly on patients with ischemic heart disease, and information is limited on the types of patient with CHD who benefit from CMR investigation, and in what ways. Therefore the aim of this study was to answer the questions: What type of patients were studied by CMR in a centre specializing in paediatric and adult CHD management? What questions were asked, which protocols were used and were the questions successfully answered? To answer these questions, we conducted a cohort study of all 362 patients that received routine clinical CMR during 2007 at the Department of Paediatric Cardiology and Congenital Heart Disease at the Deutsches Herzzentrum München.

**Results:**

Underlying diagnosis was in 33% Fallot's tetralogy, 17% aortic coarctation, 8% Ebstein's disease, 6% Marfan's disease, 4% single ventricle with Fontan-like circulation, and 32% others. Median age was 26 years (7 days – 75 years). Ventricular volumes were assessed in 67% of the patients; flow in 74%; unknown anatomy only in 9%; specific individual morphology of known anatomy in 83%; myocardial fibrosis in 8%; stress-induced myocardial perfusion defects in 1%. Only in 3% of the cases the question could not be fully answered.

**Conclusion:**

Contrary to common belief, routine CMR of patients with CHD was not requested to address global anatomical questions so much as to clarify specific questions of morphology and function of known anatomy. The CMR protocols used differed markedly from those widely used in patients with ischemic heart disease.

## Background

Moderate to severe forms of congenital heart disease (CHD) are found in about 6 of 1,000 live births[1]. All forms of CHD represent 7 to 19 of 1,000 live births [1-3]. As a result of the success of paediatric cardiology and cardiac surgery over the last three decades, there will shortly be more adults than children with CHD[4]. There are currently estimated to be at least 120,000 adults in Germany with CHD and this number is expected to rise by about 5,500 per year[5]. The number of patients with complex forms of CHD are expected to rise by about 50% within the next few years[6]. A vital component to the multidisciplinary management of the CHD patient is Cardiovascular Magnetic Resonance (CMR). CMR of patients with CHD has become routine practice during the last few years[7]. However, the majority of clinically used CMR protocols focus on adults with ischemic heart disease and relatively little information is available on protocols for patients with CHD[8,9]. Although general guidelines for CMR of patients with CHD have been developed[7,9,10], guidelines addressing the range of types of CHD have not. Nor has the range of CHD questions addressed by routine clinical CMR been recorded in paediatric and adult groups, or the duration of imaging, the place of sedation or anaesthesia, or the contribution of contrast agent. And most importantly, the clinical value or the ability to answer the question asked is unknown. Therefore the aim of this study was to answer the questions: What type of patients were studied by CMR in paediatric and adult CHD? What questions were asked, which protocols were used and were the questions successfully answered? To address these questions we analyzed the entire cohort of patients receiving a routine clinical cardiac study in a CMR-scanner in a large output center dedicated to paediatric and adult CHD during the year 2007.

## Results

The main underlying diagnoses were as follows: 121 patients (33%) had Fallot-like hemodynamics after correction (Fallot's tetralogy, absent pulmonary valve, double outlet right ventricle, and pulmonary atresia with or without a ventricular septal defect status post correction). 61 patients (17%) had aortic coarctation and/or aortic arch anomalies (native, status post surgery, or status post surgery and catheter intervention). 30 patients (8%) had Ebstein's disease (native or status post surgery). 23 patients (6%) had Marfan's syndrome or other types of aortic dilatation (native or status post surgery). 16 patients (4%) had Fontan-like circulation (different types of right atrium to pulmonary artery anastomosis or total cavopulmonary connection with intracardiac or extracardiac tunnel). 16 patients (4%) had congenital aortic valve or supravalvular aortic stenosis (native or status post aortic valve dilatation or Ross operation). The remaining 95 patients (28%) had other underlying diagnoses [see additional file 1].

Median age for the entire cohort was 26 years (7 days – 75 years). Median weight was 64 kg (2.3 – 122 kg).

Median scan duration for the whole cohort was 47 min (2 – 110 min).

The majority of patients (n = 347, 96%) did not require sedation or intubation. Four patients (1%) required sedation without intubation. The median age of these four patients was 40 days (7 days – 2.4 months). The median scan duration of these four patients was 35 min (16 – 49 min). Eleven patients (3%) required anesthesia with intubation. The median age of these eleven patients was 3.3 years (15 days – 5.8 years). The median scan duration of these eleven patients was 38 min (20 – 131 min).

228 patients (63%) received contrast agent.

The questions asked were successfully answered in 350 patients (97%). The questions asked were only partially answered in five patients (1%), and not answered in seven patients (2%).

The reasons for an unsuccessful CMR-study were coil-artefacts (n = 3), arrhythmia (n = 2), unreliable ECG- or pulse-wave-triggering (n = 2), stent-artefacts in a patient with coarctation of the aorta (n = 1), impossible positioning of a patient with extreme scoliosis (n = 1), a previously undetected retrocardial surgical needle (n = 1), unconclusive flow and morphology results (n = 1), and claustrophobia (n = 1).

Ventricular volumes were assessed in 241 patients (67%).

Flow was assessed in 267 patients (74%). Median number of flow measurements per patient was two (0 – 6). One flow measurement was carried out in 30 patients (8%), two flow measurements in 72 patients (20%), three flow measurements in 18 patients (5%), four flow measurements in 132 patients (36%), five flow measurements in eight patients (2%), and six flow measurements in seven patients (2%).

Unknown anatomy was assessed only in 31 patients (9%).

Specific individual morphology of known anatomy was assessed in 299 patients (83%).

Myocardial fibrosis was assessed only in 29 patients (8%).

Stress-induced myocardial perfusion defects were assessed only in three patients (1%).

## Methods

The study cohort consisted of all 362 patients that received routine clinical CMR between Januray, 1^st ^and December, 31^st^, 2007 at the Department of Paediatric Cardiology and Congenital Heart Disease at the Deutsches Herzzentrum München.

A standard cardiac 1.5 Tesla MRI-scanner and a standard cardiac 12-channel coil were used for all patients (MAGNETOM Avanto^®^, Siemens Healthcare, Erlangen, Germany). A standard MR-compatible anaesthesia monitor (Invivo, Orlando, USA) was used to continuously monitor ECG, blood pressure, transcutaneous saturation, exhaled CO_2 _of all patients during anaesthesia with intubation or sedation without intubation.

All CMR reports of the entire cohort were retrospectively reviewed.

The following parameters were analysed: diagnosis, age, weight in kg, use of sedation or intubation, scan-duration defined as time between first and last scan, use of contrast agent (gadopentetate dimeglumine, Magnevist^®^, Bayer Health Care, Leverkusen, Germany), sequences used and the ability to answer the questions asked.

According to the question asked, as documented on the CMR report, we defined six types of questions to be answered: (1) Ventricular volumes, e.g. right ventricular end diastolic volume for the evaluation of patients with repaired Fallot's tetralogy. (2) Flow through a vessel, e.g. forward and backward flow through the pulmonary artery for calculation of pulmonary regurgitation for the evaluation of patients with repaired Fallot's tetralogy. (3) Unknown anatomy, e.g. search for veno-venous collaterals late after Fontan-type surgery. (4) Specific individual morphology of known anatomy, e.g. imaging of homograft morphology for planning percutaneous pulmonary valve implantation. (5) Presence of myocardial fibrosis, e.g. status post coronary event. (6) Presence of stress-induced myocardial perfusion defect, e.g. evaluation of chest pain status post coronary artery surgery. By grouping the sequences used according to the question to be answered, we therefore defined six protocols. (1) ventricular volume protocol (using multiphased, multislice cine SSFP sequences in axial or short axis orientation, temoral resolution: 20–40 ms, slice thickness: 4–6 mm), (2) flow protocol (using phase-encoded multiphased sequences, free breathing, retrospective ECG-gating, velocity encoding 150–500 cm/s), (3) unknown anatomy protocol (using 3-dimensional navigator- and ECG-triggered SSFP sequences, contrast enhanced angiography, and multiphased cine SSFP sequences), (4) specific individual morphology of known anatomy protocol (using 3-dimensional navigator- and ECG-triggered SSFP sequences, contrast enhanced angiography, and multiphased cine SSFP sequences), (5) myocardial fibrosis protocol (using Late Gadolinium Enhancement sequences), and (6) stress-induced myocardial perfusion defect protocol (using adenosin-stress-perfusion sequences).

The question asked, as documented on the CMR report, was defined as successfully answered according to the conclusion, as documented on the CMR report. If only parts of the question asked were successfully answered (e.g. successful volume analysis but unsuccessful flow analysis), the question was defined as partially answered. If during the retrospective analysis there was a doubt, that the answer asked was not successfully answered, the question was defined as not answered.

## Discussion

The results of this review of clinical practice study show that the largest patient group (33% of all patients) studied in a CMR system dedicated largely to CHD were young adults with Fallot-like hemodynamics after repair (Fallot's tetralogy, absent pulmonary valve, double outlet right ventricle, and pulmonary atresia with or without a ventricular septal defect status post correction). The second largest group (17%) were young adults with coarctation of the aorta or other aortic arch anomalies before or after surgery. These two groups accounted for half of the patients. The other half consisted of a heterogenous group of patients [see additional file 1].

The results of this study show that in two thirds of the cases quantification of ventricular volumes was amongst the questions asked [see additional file 1] and in three quarters, quantification of blood flow was requested. This contrasts with CMR imaging in patients with acquired heart disease [7,9] who, except in the presence of valvular heart disease, rarely require quantification of blood flow[8,9].

Furthermore, the results show that investigation of myocardial fibrosis or stress-induced myocardial perfusion defects was rarely requested, again in contrast to patients with acquired heart disease [8,9]. However, this may reflect our own experience [11], that fibrosis is not a common finding in patients with CHD.

Another important result of this study is that unknown anatomy was rarely (9%) a question asked in the study cohort. However, specific individual morphology of known anatomy was the most commonly asked question (83%). These findings support the previously stated hypothesis [12-14] that CMR in paediatric and adult CHD is a tool for answering questions regarding specific individual morphology and hemodynamic function of *known *anatomy. This is in contrast to the perception that the primary goal of CMR in paediatric and adult CHD is to describe the location and anatomy of a congenital heart defect [15-18].

We also found that the overwhelming majority (97%) of all cases the questions asked were successfully answered by the CMR study.

In our series, one CMR-study could not be completed due to a previously undetected retrocardial surgical needle. After positioning the patient in the magnet, the scout sequences showed a large artifact for which no external explanation was found. A standard chest x-ray revealed a retrocardiac surgical needle. A retrospective review of the report of the operation 24 years previously in another hospital showed that the missing needle had been mentioned but could not be retrieved during the operation.

An important limitation of our study is that the study cohort reflects the specific situation in our center, the findings may not necessarily be applicable to other CHD centers. At our hospital (Deutsches Herzzentrum München) all paediatric patients with cardiac problems and all patients with CHD regardless of age are treated in a single Department of Paediatric Cardiology and Congenital Heart Disease, whereas it may be common practice for paediatric and adult patients to be managed in different departments. Our center may also differ from other centers in two further points. Firstly, in the larger Munich area close pre- and postnatal echocardiography is available and diagnosis and anatomy were usually well established by the first visit. Secondly, our patients are closely followed up throughout life and their anatomy continues to be well documented. Potential further differences to other centers may be related to our CMR service. Our hospital has one 1.5T MRI-scanner (MAGNETOM Avanto^®^, Siemens Healthcare, Erlangen, Germany). The scanner was used by all three departments of our hospital (Department of Paediatric Cardiology and Congenital Heart Disease, Department of Cardiology, and Department of Cardiac Surgery). The scanner was used for clinical and research questions. The scanner was used for cardiac imaging and also non-cardiac imaging (e.g. pre-/post OP imaging of the brain). The patients were booked at intervals through the day depending on the estimated scan time, all patients from the Department of Paediatric Cardiology and Congenital Heart Disease (cohort of this study) being booked hourly. On each working day two patients from the Department of Paediatric Cardiology and Congenital Heart Disease were booked well in advance (our current waiting list is three to four months). Up to two further cases a day from the Department of Paediatric Cardiology and Congenital Heart Disease could be scheduled at short notice to address more urgent questions. The cohort of this study (n = 362) represented 26% of all patients scanned during 2007. Of note, the scan time of patients in this cohort was longer than most other patient cohorts, therefore the estimated total scan duration of the cohort of this study represented about 40% of total scan time during 2007. Postprocessing the data was also time-consuming, taking about one hour of postprocessing- and reporting-time per patient and requiring expertise in both CHD and CMR. Our set-up consisted of one expert in CHD and CMR (SF or HS), a trainee (AS or CB), and a technician. The expert in CHD and CMR planned the study, was responsible for the patient information and consent form, was present during the whole study, and wrote the report. The trainee was an interested beginner in CHD and well-trained in volume and flow analysis. The trainee analysed the volume and flow studies on a separate work station during the on-going study. The technician rotated to the scanner from a pool of technicians with variable knowledge of CHD and CMR. The results of the studies were presented the following working day by the expert in CHD and CMR on a daily conference including presentation of other imaging modalities. For these conferences the expert in CHD and CMR presented the data and the images using a dedicated work station and projector.

Only a relatively small subgroup of patients (n = 15/362, 4%) required anaesthesia with intubation, or sedation without intubation. Therefore, the relative safety of anaesthesia with intubation or sedation without intubation cannot be deduced from the numbers available. Not all clinicians would necessarily accept that sedation of non-intubated patients with CHD is acceptably safe when imaging in the confines of a magnet. On the other hand however, not all clinicians would necessarily accept that intubation of a patient is justified for a short non-invasive study that does not necessarily require interruption of free breathing. The decision to carry out CMR under anaesthesia with intubation or sedation without intubation was based on three points at our center: the need for acquisition during interrupted breathing, the expected compliance of the patient and the preference of the anaesthesiologist. The expert in CHD and CMR (SF or HS) decided first after speaking with the parent of the patient if the CMR could be carried out without any form of anaesthesia or sedation. If this was not the case, he decided, depending on the need for breath holds and the anticipated length of the study, if anaesthesia with intubation or sedation without intubation was needed. He then reached a final decision with the responsible anaesthesiologist.

## Conclusion

Taken together, the results of this study show that patients referred to CMR in paediatric and adult CHD had already been evaluated anatomically, and the main reasons for referral for CMR were questions regarding specific individual morphology and function of known anatomy.

This cohort study of patients receiving a routine clinical CMR study in a scanner dedicated to paediatric and adult CHD in 2007 may provide a basis for hospital planners and the CMR industry to understand the needs of patients with CHD.

## Competing interests

Sohrab Fratz has received speaker fees from Siemens Healthcare, Erlangen, Germany. All other authors have no conflict of interest.

## Authors' contributions

SF planned, designed and conducted the study, carried out all CMR studies, analyzed and interpreted the entire data, and wrote the draft and the final version of the manuscript. JH took part in the conception and design of the study and reviewed the drafts of the manuscript for important intellectual content. AS took part in the conception and design of the study, in the evaluation of a substantial number of the CMR studies, reviewed the drafts of the manuscript for important intellectual content.

CB took part in the conception and design of the study, in the evaluation of a substantial number of the CMR studies, reviewed the drafts of the manuscript for important intellectual content. EH took part in the conception and design of the study, assisted in the evaluation of a few CMR studies, reviewed the drafts of the manuscript for important intellectual content. SM took part in the conception and design of the study, reviewed the drafts of the manuscript for important intellectual content.

HS took part in the conception and design of the study, reviewed the drafts of the manuscript for important intellectual content. All authors have seen and approved the final version of the manuscript.

## Supplementary Material

Additional file 1 Patient's demographics, protocols used, questions asked and contributions made. no further description. Click here for file
