# Supplementary material for: Routine clinical cardiovascular magnetic resonance in paediatric and adult congenital heart disease: patients, protocols, questions asked and contributions made
Source: J Cardiovasc Magn Reson. 2008 Oct 17;10(1):46. doi: 10.1186/1532-429X-10-46 (PMC2579426; doi:10.1186/1532-429X-10-46)
Supplement: Additional file 1 — Patient's demographics, protocols used, questions asked and contributions made. no further description. [file 1532-429X-10-46-S1.doc]

|  |  |  |  | **p r o t o c o l s u s e d** | | | | | |  |  |  |  |
| --- | --- | --- | --- | --- | --- | --- | --- | --- | --- | --- | --- | --- | --- |
| **underlying diagnosis** | number of pts | **median age (range)** | **median weight (range)** | **volume** | **flow studies per pt** | **unknown anatomy** | **specific individual morphology** | **myocardial fibrosis** | **stress-induced myocardial perfusion defect** | **scan duration (range)** | **intubation / sedation** | **pts recieving contrast agent** | **successfully answered question** |
|  | **[n (%)]** | **[yrs]** | **[kg]** | **[n (%)]** | **median (range)** | **[n (%)]** | **[n (%)]** | **[n (%)]** | **[n (%)]** | **[hrs:min]** | **[n (%)]** | **[n (%)]** | **[n (%)]** |
| Fallot-like hemodynamics | 121 (33) | 23  (8 - 57) | 60  (23 – 106) | 111 (92) | 4 (0 - 6) | 5 (4) | 106 (88) | 4 (3) | 0 | 0:54  (0:02 - 1:50) | 0 | 103 (85) | 118 (98) |
| coarctation of the aorta and aortic arch anomaly | 61 (17) | 28 (0.02 - 71) | 65  (2 - 108) | 13 (21) | 1 (0 - 4) | 7 (11) | 59 (97) | 0 | 0 | 0:36  (0:14 - 1:31) | 5 (8) | 44 (72) | 59 (97) |
| Ebstein´s disease | 30 (8) | 28  (2 - 75) | 61  (12 - 94) | 27 (90) | 2 (0 - 4) | 0 | 5 (17) | 1 (3) | 0 | 0:39  (0:15 - 1:20) | 1 (3) | 3 (10) | 29 (97) |
| Marfan´s syndrome and other aortic dilatation | 23 (6) | 36  (10- 54) | 73  (33 – 103) | 3 (13) | 0 (0 - 3) | 1 (4) | 23 (100) | 1 (4) | 0 | 0:36  (0:08 - 1:30) | 0 | 14 (61) | 23 (100) |
| Fontan-like circulation | 16 (4) | 23  (4 - 53) | 63  (17 – 112) | 9 (56) | 4 (0 - 6) | 4 (25) | 13 (81) | 0 | 1 (6) | 0:44  (0:20 - 1:26) | 2 (13) | 4 (25) | 16 (100) |
| congenital aortic valve and supravalvular aortic stenosis | 16 (4) | 28  (17 - 49) | 80  (50 - 94) | 8 (50) | 1 (0 - 6) | 2 (13) | 15 (94) | 2 (13) | 1 (6) | 0:40  (0:12 - 1:08) | 0 | 9 (56) | 16 (100) |
| myocardial disease (ARVC, HOCM, M.Fabry, Myocarditis) | 16 (4) | 29  (0.7 - 68) | 75  (4 - 104) | 13 (81) | 0 (0 - 2) | 1 (6) | 12 (75) | 11 (67) | 0 | 0:55  (0:05 - 1:26) | 2 (13 %) | 15 (94) | 15 (94) |
| TGA s/p atrial switch operation | 14 (4) | 31  (22 - 39) | 69  (50 – 101) | 12 (86) | 2 (0 - 4) | 0 | 14 (100) | 2 (14) | 0 | 0:50  (0:25 - 1:24) | 0 | 4 (29) | 14 (100) |
| non-Fontan univentricular heart circulation | 10 (3) | 19  (0.2 - 32) | 43  (3 - 73) | 9 (90) | 4 (0 - 6) | 1 (10) | 7 (70) | 0 | 0 | 0:47  (0:20 - 1:26) | 2 (20) | 4 (40) | 10 (100) |
| s/p truncus arteriosus repair | 8 (2) | 22  (20 - 41) | 58  (55 – 122) | 6 (75) | 4 (0 - 6) | 1 (13) | 8 (100) | 0 | 0 | 0:51  (0:21 - 1:10) | 0 | 6 (75) | 8 (100) |
| Eisenmenger´s disease | 7 (2) | 37  (16 - 52) | 61  (56 - 72) | 5 (71) | 2 (0 - 4) | 1 (14) | 3 (43) | 1 (14) | 0 | 0:32  (0:16 - 1:22) | 0 | 2 (29) | 7 (100) |
| TGA s/p arterial switch operation | 6 (2) | 16  (1 - 31) | 42  (11 - 74) | 4 (67) | 1 (0 - 4) | 0 | 4 (67) | 1 (17) | 1 (17) | 0:49  (0:37 - 1:35) | 1 (17) | 3 (50) | 6 (100) |
| TGA s/p Rastelli operation | 5 (1) | 14  (11 - 45) | 52  (32 - 83) | 5 (100) | 4 (4 - 4) | 0 | 5 (100) | 0 | 0 | 0:42  (0:31 - 0:55) | 0 | 2 (40) | 5 (100) |
| atrial septal defect or sinus venosus defect | 4 (1) | 31  (11 - 44) | 66  (43 - 82) | 4 (100) | 2 (2 - 2) | 1 (25) | 3 (75) | 0 | 0 | 0:46  (0:19 - 0:55) | 0 | 1 (25) | 4 (100) |
| congenitally corrected transposition of the great arteries | 4 (1) | 57  (34 - 65) | 88  (59 – 100) | 4 (100) | 4 (0 - 5) | 1 (25) | 3 (75) | 1 (25) | 0 | 0:53  (0:46 - 1:14) | 0 | 3 (75) | 4 (100) |
| before ablation, no congenital heart disease | 3 (1) | 13  (6 - 15) | 45  (20 - 50) | 3 (100) | 0 | 0 | 3 (100) | 1 (33) | 0 | 0:50  (0:28 - 1:09) | 0 | 1 (33) | 3 (100) |
| cardiac tumor | 3 (1) | 44  (9 - 66) | 93  (28 - 99) | 1 (33) | 0 | 0 | 3 (100) | 2 (67) | 0 | 1:23  (1:10 - 1:28) | 0 | 2 (67) | 3 (100) |
| complete atrioventricular defect | 3 (1) | 15  (12 - 30) | 40  (40 - 60) | 2 (67) | 4 (2 - 4) | 1 (33) | 3 (100) | 0 | 0 | 0:55  (0:13 - 1:32) | 0 | 1 (33) | 3 (100) |
| ventricular septal defect | 3 (1) | 21  (7 - 34) | 44  (19 - 68) | 1 (33) | 0 (0 - 4) | 2 (67) | 3 (100) | 0 | 0 | 0:28  (0:18 - 0:45) | 0 | 1 (33) | 3 (100) |
| left ventricular diverticula or right atrium aneurysm | 2 (1) | 19  (5-32) | 52  (21 - 82) | 1 (50) | 0 | 0 | 2 (100) | 1 (50) | 0 | 0:35  (0:33 - 0:38) | 1 (50) | 2 (100) | 2 (100) |
| left pulmonary artery agenesia or pulmonary sling | 2 (1) | 19  (0.2 - 38) | 37  (5 - 69) | 1 (50) | 3 (2-4) | 1 (50) | 1 (50) | 0 | 0 | 0:44  (0:27 - 1:27) | 1 (50) | 2 (100) | 2 (100) |
| partial anomolous pulmonary vein connection | 1 (0) | 19 | 61 | 0 | 0 | 1 (100) | 1 (100) | 0 | 0 | 1:22 | 0 | 1 (100) | 1 (100) |
| Kawasaki´s disease | 1 (0) | 24 | 50 | 0 | 0 | 0 | 1 (100) | 1 (100) | 0 | 0:40 | 0 | 1 (100) | 1 (100) |
| constrictive pericarditis | 1 (0) | 27 | 60 | 1 (100) | 2 | 0 | 1 (100) | 0 | 0 | 0:41 | 0 | 0 | 1 (100) |
| single ventricle post septation | 1 (0) | 36 | 78 | 1 (100) | 2 | 0 | 0 | 0 | 0 | 0:28 | 0 | 0 | 1 (100) |
| pulmonary artery ectasia | 1 (0) | 14 | 45 | 0 | 0 | 1 (100) | 1 (100) | 0 | 0 | 0:43 | 0 | 0 | 1 (100) |
| **total cohort** | **362 (100)** | **26**  **(0.02 - 75)** | **64**  **(2 - 122)** | **241 (67)** | **2 (0 - 6)** | **31 (9)** | **299 (83)** | **29 (8)** | **3 (1)** | **0:47**  **(0:02 - 1:50)** | **15 (4)** | **228 (63)** | **350 (97)** |
